# Supplementary material for: To flea or not to flea: survey of UK companion animal ectoparasiticide usage and activities affecting pathways to the environment
Source: PeerJ. 2023 Aug 4;11:e15561. doi: 10.7717/peerj.15561 (PMC10405796; doi:10.7717/peerj.15561)
Supplement: Supplemental Information 1 [file peerj-11-15561-s001.docx]

**Supplementary data file 2**

**Demographics**

*Table S1. Demographic data for survey respondents. n = number of responses, % = percentage of responses.*

| **Demographic** | **Variable** | **n** | **%** |
| --- | --- | --- | --- |
| **Gender** | Female | 849 | 84.1 |
|  | Male | 149 | 14.7 |
|  | Agender/Prefer not to say | 11 | 1.1 |
| **Age (years)** | 18-24 | 55 | 5.5 |
|  | 25-34 | 208 | 20.6 |
|  | 35-44 | 199 | 19.7 |
|  | 45-54 | 272 | 27.0 |
|  | 55-65 | 205 | 20.3 |
|  | Over 65 | 70 | 6.9 |
| **Region** | East | 91 | 9.0 |
|  | East Midlands | 74 | 7.3 |
|  | London | 37 | 3.7 |
|  | North East | 33 | 3.3 |
|  | North West | 111 | 11.0 |
|  | Northern Ireland | 12 | 1.2 |
|  | Scotland | 70 | 6.9 |
|  | South East | 253 | 25.1 |
|  | South West | 148 | 14.7 |
|  | Wales | 58 | 5.7 |
|  | West Midlands | 69 | 6.8 |
|  | Yorkshire And The Humber | 53 | 5.3 |

**Dog ectoparasiticides**

*Table S2. Responses to the question: “During the last 12 months, what is the main flea product used on your dog?”, with active ingredients listed. Respondents that had not treated their dog in that time (n = 83) or applied an unrecognised/alternative treatment (n=14) are not included. n = number of responses, % = percentage of responses.*

| **Product** | **Active ingredients** | **n** | **%** |
| --- | --- | --- | --- |
| Advantage Spot-on | Imidacloprid | 20 | 4.1 |
| Advantix Spot-on | Imidacloprid, permethrin | 4 | 0.8 |
| Advocate Spot-on | Imidacloprid, moxidectin | 93 | 18.9 |
| FIPROtec COMBO Spot-on | Fipronil, s-methoprene | 2 | 0.4 |
| Bob Martin Clear /Clear Plus Spot-on | Fipronil/ fipronil, s-methoprene | 7 | 1.4 |
| Bravecto Spot-on | Fluralaner | 6 | 1.2 |
| Bravecto Tablets | Fluralaner | 128 | 26 |
| Capstar Tablets | Nitenpyram | 2 | 0.4 |
| Credelio Tablets | Lotilaner | 3 | 0.6 |
| Easecto Spot-on | Sarolaner | 5 | 1 |
| Effipro Spot-on | Fipronil | 5 | 1 |
| Endectrid Spot-on | Imidacloprid, moxidectin | 7 | 1.4 |
| Fipronil Spot-on generic | Fipronil / fipronil, s-methoprene | 6 | 1.2 |
| Frontline /Frontline Combo spot on | Fipronil / fipronil, s-methoprene | 52 | 10.6 |
| Itch – flea Spot-on | Fipronil, s-methoprene | 6 | 1.2 |
| Kiltix Collar | Propoxur, flumethrin | 1 | 0.2 |
| NexGard Tablet | Afoxalaner | 15 | 3 |
| NexGard Spectra Tablet | Afoxalaner, milbemycin oxime | 66 | 13.4 |
| Prinovox Spot-on | Imidacloprid, moxidectin | 8 | 1.6 |
| Seresto Collar | Imidacloprid, flumethrin | 27 | 5.5 |
| Simparica Tablets | Sarolaner | 17 | 3.5 |
| Stronghold Spot-on | Selamectin | 12 | 2.4 |
| Total |  | 492 | 100 |

**Cat ectoparasiticides**

*Table S3. Responses to the question: “During the last 12 months, what is the main flea product used on your cat?”, with active ingredients listed. Respondents that had not treated their cat in that time (n = 36) or applied an unrecognised/ alternative treatment (n=7) are not included. n = number of responses, % = percentage of responses.*

| **Product** | **Active ingredients** | **n** | **%** |
| --- | --- | --- | --- |
| Activyl Spot-on | Indoxacarb | 3 | 0.8 |
| Advantage Spot-on | Imidacloprid | 50 | 14.2 |
| Advocate Spot-on | Imidacloprid, moxidectin | 59 | 16.7 |
| Bob Martin Clear / Clear Plus Spot-on | Fipronil/ fipronil, moxidectin | 11 | 3.1 |
| Bravecto/Bravecto Plus spot on | Fluralaner/fluralaner, moxidectin | 52 | 14.7 |
| Broadline Spot-on | Fipronil, Eprinomectin, praziquantel, s-methoprene | 19 | 5.4 |
| Comfortis Tablet | Spinosad | 4 | 1.1 |
| Credelio Tablet | Lotilaner | 11 | 3.1 |
| Effipro Spot On | Fipronil, s-methoprene | 7 | 2 |
| Felisecto Plus Spot-on | Selamectin, sarolaner | 1 | 0.3 |
| Fipronil Spot-on generic | Fipronil/ fipronil, s-methoprene | 10 | 2.8 |
| Frontline /Frontline Combo Spot-on | Fipronil/ fipronil, s-methoprene | 58 | 16.4 |
| Itch – flea Spot-on | Fipronil, s-methoprene | 5 | 1.4 |
| Prinovox Spot-on | Imidacloprid, moxidectin | 4 | 1.1 |
| Program Injection | Lufenuron | 5 | 1.4 |
| Seresto Collar | Imidacloprid, flumethrin | 8 | 2.3 |
| Stronghold / Stronghold Plus Spot-on | Selamectin/ Selamectin, sarolaner | 44 | 12.5 |
| Vectra Spot-on | Dinotefuran, pyriproxifen | 2 | 0.6 |
| Total |  | 353 | 100 |

**Veterinary Advice**

*Table S4. The* frequency and percentage of responses to the question “*What advice has been given by your veterinarian regarding flea/tick treatment?”. n = number of responses, % = percentage of respondents*

| **Answer** | **n** | **%** |
| --- | --- | --- |
| Regular preventative flea/tick treatment throughout the year | 794 | 81.3% |
| Regular preventative flea/tick treatment in warmer months only | 30 | 3.1% |
| Treat only if fleas/ticks are seen | 21 | 2.1% |
| Treat only if fleas/ticks are seen, your pet has a history of being prone to flea/tick infestations, or suffers from skin allergies | 8 | 0.8% |
| Other advice given | 16 | 1.6% |
| No advice given | 108 | 11.1% |
| Total | 977 | 100 |

**Disposal of animal waste**

*Table S5. Responses to the question: “What is the main way that you dispose of your dog’s poo?”. n = number of responses, % = percentage of respondents.*

| **Disposal method** | **n** | **%** |
| --- | --- | --- |
| Bag and bin (specific dog poo bin) | 274 | 45.4 |
| Bag and bin (home council bin) | 270 | 44.8 |
| Straight in a bin (of any kind) | 26 | 4.3 |
| Bury in the ground | 12 | 2 |
| Flush down the toilet | 5 | 0.8 |
| None of the above/don't know | 15 | 2.5 |
| Total | 602 | 100 |
