# Supplementary material for: To flea or not to flea: survey of UK companion animal ectoparasiticide usage and activities affecting pathways to the environment
Source: PeerJ. 2023 Aug 4;11:e15561. doi: 10.7717/peerj.15561 (PMC10405796; doi:10.7717/peerj.15561)
Supplement: Supplemental Information 2 [file peerj-11-15561-s002.pdf]

Evaluating the use of flea products in the UK

INTRODUCTION

WHAT IS THE PURPOSE OF THIS RESEARCH?

This survey aims to shed light on the quantity and type of flea products used in the UK, and how they are used.

WHAT DOES THIS SURVEY INVOLVE?

This survey is intended for UK cat and dog owners, and you may participate if you are aged 18 or older. You will complete a short online survey about one cat or dog that has lived in your household for at least one year (unless it is a puppy or kitten). You will be asked about the frequency and type of flea products used on your pet, as well as your pet's bathing, swimming and sleeping habits and history of flea infestations. You will also be asked about yourself, and the advice you have received regarding pet flea treatment.

The survey usually takes 5-10 minutes to complete.

WHO IS CONDUCTING THIS RESEARCH?

Rose Perkins, from the School of Life Sciences at the University of Sussex. Rose is a veterinary surgeon doing a PhD on the use of pet flea treatments, through funding from the Veterinary Medicines Directorate.

DATA PROTECTION AND PRIVACY

Participation in this survey is completely anonymous and voluntary, and participants may withdraw at any time. No personal or identifying information will be collected and you can ask for your data to be destroyed and/or removed from the project until it is no longer practical to do so. Your data will be processed in accordance with the UK's Data Protection Act 2018 and the General Data Protection Regulations (GDPR). Data collection is taking place through a secure online survey platform, and all data will be

stored securely on a University managed storage system. IP address won't be collected during this survey. The results of this research may be published in academic journals

or other media, presented at various fora, discussed in the mass media, or shared for research purposes. This data collection and research project is conducted in accordance with the University of Sussex data and ethics guidelines for surveys: http://www.sussex.ac.uk/library/researchdatamanagement/create/dataprotectionandethics
This study has been approved by the Sciences & Technology Cross-Schools Research Ethics Committee. The project reference number is ER/AT459/3. If you have any ethical concerns, please contact the ethics chair (crecscitec@sussex.ac.uk).
The University of Sussex has insurance in place to cover its legal liabilities in respect of this study.

WHO CAN I CONTACT IF I HAVE QUESTIONS?

Please feel free to contact Rose Perkins (rp442@sussex.ac.uk) at the School of Life Sciences, Sussex University if you have any questions.

Thank you for taking the time to participate in this research.

I agree to take part in the above University of Sussex Research Project:

- Yes
- No

Confirm requirements

Please confirm that you are 18 or older, and that your answers relate to one cat or dog that has been in your household for at least one year, unless it is a kitten or puppy.

- Yes
- No

Pet species

My animal is a:

- Cat
- Dog

Dogs part 1

Please indicate the size of your dog:

- Miniature (below 4 kg). Example: Chihuahua
- Small (4-10kg). Example: Jack Russell
- Medium (10-20 kg). Example: Cocker spaniel
- Large (20-40kg). Example: Labrador
- Extra large (over 40 kg). Example: Great Dane

If known, please indicate (in kg) the approximate weight of your dog (number only).

How old is your dog in years? (Best estimate)

- <1
- 1
- 2
- 3
- 4
- 5
- 6
- 7
- 8
- 9
- 10
- 11
- 12
- 13
- 14
- 15
- 16
- 17
- 18

- 19
- 20
- >20

In the last 12 months, have you administered a flea treatment to your dog?

- Yes
- No
- Don't know

What was your main reason for administering a flea treatment to your dog?

- To prevent fleas (or other pests)
- In response to a flea infestation
- In response to other pests (eg ticks)
- Other reason
- Don't know

In the last 12 months, have you used a natural / alternative flea remedy on your dog?

- Yes
- No
- Don't know

Natural/alternative flea remedies

If known, please specify the natural/alternative flea remedy that you have used

Flea treatment dogs

During the last 12 months, which flea product/s have you used on your dog?

- Advocate
- Activyl
- Advantage
- Advantix
- Bravecto tablet

- Bravecto spot on
- Bob Martin Clear /Clear Plus (Fipronil)
- Capstar
- Comfortis
- Credelio
- Effipro Spot On
- Endectrid
- Fipnil/ Fipralone/ Fiprene/ Fiprex/ Fiproclear/ Fiprofile/ Fipronil/ Fiprotec/ Fiproxole/
- Fleanil/ Fleascreen/ Flevox/ Flick/ Fyperix
- Front line or Front line Combo spot on
- Imidacloprid/ Imidaflea/ Imidaspot
- Itch - flea
- NexGard
- NexGard Spectra
- Prinovox
- Program
- Simparica
- Stronghold
- Seresto collar
- Don't know
- Other

During the last 12 months, what is the main flea product used on your dog?

- Advocate
- Activyl
- Advantage
- Advantix
- Bravecto tablet
- Bravecto spot on
- Bob Martin Clear /Clear Plus (Fipronil)
- Capstar
- Comfortis
- Credelio
- Endectrid
- Effipro Spot On

- Fipnil/ Fipralone/ Fiprene/ Fiprex/ Fiproclear/ Fiprofile/ Fipronil/ Fiprotec/ Fiproxole/ Fleanil/ Fleascreen/ Flevox/ Flick/ Fyperix
- Front line or Front line Combo spot on
- Imidacloprid/ Imidaflea/ Imidaspot
- Itch - flea
- NexGard
- NexGard Spectra
- Prinovox
- Program
- Simparica
- Stronghold
- Seresto collar
- Don't know
- Other

In the last 12 months, approximately how many times have you administered a flea treatment to your dog?

- 1
- 2
- 3
- 4
- 5
- 6
- 7
- 8
- 9
- 10
- 11
- 12
- Don't know

Where do you usually purchase flea products for your dog?

- From a veterinary practice
- From a petshop
- From a supermarket

- Online
- Dog show event or similar
- Other type of shop - pharmacy/agricultural/equestrian/other
- None of the above

If you use a spot-on flea product on your dog, do you stroke your dog in the 24 hours after administering flea treatment? (Skip if not applicable)

- Yes
- No
- Don't know

If you apply a spot-on flea product or flea collar to your dog, are you aware of product warnings regarding swimming or bathing after flea treatment? (Skip if not applicable)

- Yes
- No

If the above answer is yes, do you follow the product warnings regarding swimming or bathing after flea treatment? (Skip if not applicable)

- Yes
- No

In the last 12 months, have you seen or suspected any side effects to flea treatments in your dog?

- Yes
- No
- Maybe

Dogs (general)

In the last 12 months, have you suspected that your dog has fleas?

- Yes
- No
- Don't know

In the last 12 months, have you seen fleas on your dog or in your household?

- ◯ Yes
- ◯ No
- ◯ Don't know

Monthly occurrence of fleas (dogs)

Approximately what month/s of the year did you see fleas on your dog or in your household?

- ☐ January
- ☐ February
- ☐ March
- ☐ April
- ☐ May
- ☐ June
- ☐ July
- ☐ August
- ☐ September
- ☐ October
- ☐ November
- ☐ December
- ☐ Don't know

Ticks

In the last 12 months, have you seen ticks on your dog?

- ◯ Yes
- ◯ No
- ◯ Don't know

Approximately what month/s of the year did you see ticks on your dog?

- ☐ January
- ☐ February

- [ ] March
- [ ] April
- [ ] May
- [ ] June
- [ ] July
- [ ] August
- [ ] September
- [ ] October
- [ ] November
- [ ] December
- [ ] Don't know

Dogs part 2

In the last 12 months, approximately how many times have you treated your dog for worms?

- ○ None
- ○ 1
- ○ 2
- ○ 3
- ○ 4
- ○ 5
- ○ 6
- ○ 7
- ○ 8
- ○ 9
- ○ 10
- ○ 11
- ○ 12
- ○ Don't know

Where does your dog sleep? Select all that apply

- [ ] In their own bed
- [ ] On the furniture
- [ ] On a family member's bed

- [ ] On the floor
- [ ] Outdoors kennel/building
- [ ] In a crate
- [ ] Other/ don't know

What is your dog's most regular sleeping place? Select one

- ○ In their own bed
- ○ On the furniture
- ○ On a family member's bed
- ○ On the floor
- ○ Outdoors kennel/building
- ○ In a crate
- ○ Other / don't know

Approximately how frequently do you wash your dog's bedding?

- ○ Every week or more
- ○ Every 2 weeks
- ○ Every month
- ○ Every 3 months
- ○ Every 6 months
- ○ Once a year
- ○ Less than once a year
- ○ Never
- ○ Don't know

Approximately how frequently does your dog sit on your lap or rest against you?

- ○ Every day
- ○ Every 2 days
- ○ Every week
- ○ Every month
- ○ Every 6 months
- ○ Once a year
- ○ Less than once a year
- ○ Never

- Don't know

Approximately how frequently do you wash or hose down your dog?

- Every week or more
- Every 2 weeks
- Every month
- Every 3 months
- Every 6 months
- Once a year
- Less than once a year
- Never
- Don't know

Where is your dog usually bathed or washed down? (Skip if not applicable)

- In a bath, sink or shower
- Outdoors
- At the groomers

Aproximately how frequently does your dog enter into or swim in lakes or rivers?

- Every week or more
- Every 2 weeks
- Every month
- Every 3 months
- Every 6 months
- Once a year
- Less than once a year
- Never
- Don't know

On average, for how long is your dog walked every day?

- 10 minutes
- 30 minutes
- 1 hour

- 2 hours
- 3 hours
- 6 hours or more
- My dog doesn't go for walks
- Don't know

Approximately how much time does your dog spend outdoors each day (including walks)?

- None
- 10 minutes
- 30 minutes
- 1 hour
- 2 hours
- 3 hours
- 6 hours
- 12 hours
- Over 12 hours
- Don't know

In what type of area do you usually walk your dog? (Skip if not applicable)

- Mainly on unpaved surfaces like field margins, forest tracks or the beach
- Mainly on paved surfaces
- An equal mixture of the above

How often do you walk your dog when it is raining? (Skip if not applicable)

- Never
- Occasionally, but I try to avoid this
- Yes, I will walk my dog in light drizzle
- Yes, I walk my dog even if it is raining heavily and my dog gets completely wet

On a scale of 0-10, with 0 being 'never', and 10 being 'always', how often do you dispose of your dog's poo (ie not left on the ground)?

- 1

- 2
- 3
- 4
- 5
- 6
- 7
- 8
- 9
- 10
- Don't know

How do you dispose of your dog's poo? Select all that apply.

- [ ] Bag and bin (specific dog poo bin)
- [ ] Bag and bin (home council bin)
- [ ] Straight in a bin (of any kind)
- [ ] Flush down the toilet
- [ ] Bury in the ground
- [ ] None of the above/don't know

On a scale of 0-10, with 0 being 'never', and 10 being 'always', how often do you dispose of your dog's poo (ie not left on the ground)? 0

- 1
- 2
- 3
- 4
- 5
- 6
- 7
- 8
- 9
- 10
- Don't know

How do you dispose of your dog's poo? Select all that apply.

- ☐ Bag and bin (specific dog poo bin)
- ☐ Bag and bin (home council bin)
- ☐ Straight in a bin (of any kind)
- ☐ Flush down the toilet
- ☐ Bury in the ground
- ☐ None of the above/don't know

Cats

Please indicate the size of your cat

- ○ Small (below 4kg)
- ○ Large (above 4kg)

If known, please indicate (in kg) the approximate weight of your cat (number only)

____

How old is your cat in years? (Best estimate)

- ○ <1
- ○ 1
- ○ 2
- ○ 3
- ○ 4
- ○ 5
- ○ 6
- ○ 7
- ○ 8
- ○ 9
- ○ 10
- ○ 11
- ○ 12
- ○ 13
- ○ 14
- ○ 15
- ○ 16
- ○ 17

- 18
- 19
- 20
- 21
- 22
- 23
- 24
- >24

In the last 12 months, have you administered a flea treatment to your cat?

- Yes
- No
- Don't know

Cat flea treatments

What was your main reason for administering a flea treatment to your cat?

- To prevent fleas (or other pests)
- In response to a flea infestation
- In response other pests (eg ticks)
- Other reason
- Don't know

In the last 12 months, have you used a natural / alternative flea remedy on your cat?

- Yes
- No
- Don't know

Natural / alternative flea remedies (cats)

If known, please specify the natural/alternative flea remedy that you have used

Cat flea treatments

During the last 12 months, which flea product/s have you used on your cat?

- [ ] Advocate
- [ ] Activyl
- [ ] Advantage
- [ ] Bravecto
- [ ] Broadline
- [ ] Bob Martin Clear / Clear Plus (Fipronil)
- [ ] Capstar
- [ ] Comfortis
- [ ] Credelio
- [ ] Effipro Spot On
- [ ] Endectrid
- [ ] Fipnil/ Fipralone/ Fiprene/ Fiprex/ Fiproclear/ Fiprofile/ Fipronil/ Fiprotec/ Fiproxole/ Fleanil/ Fleascreen/ Flevox/ Flick/ Fyperix
- [ ] Front line or Front line Combo
- [ ] Imidacloprid/ Imidaflea/ Imidaspot
- [ ] Itch - flea
- [ ] Prinovox
- [ ] Program injection
- [ ] Program oral suspension
- [ ] Stronghold
- [ ] Seresto collar
- [ ] Vectra
- [ ] Don't know
- [ ] Other:

During the last 12 months, what is the main flea product used on your cat?

- ○ Advocate
- ○ Activyl
- ○ Advantage
- ○ Bravecto
- ○ Broadline
- ○ Bob Martin Clear / Clear Plus (Fipronil)
- ○ Capstar
- ○ Comfortis

- Credelio
- Effipro Spot On
- Endectrid
- Fipnil/ Fipralone/ Fiprene/ Fiprex/ Fiproclear/ Fiprofile/ Fipronil/ Fiprotec/ Fiproxole/ Fleanil/ Fleascreen/ Flevox/ Flick/ Fyperix
- Front line or Front line Combo
- Imidacloprid/ Imidaflea/ Imidaspot
- Itch - flea
- Prinovox
- Program injection
- Program oral suspension
- Stronghold
- Seresto collar
- Vectra
- Don't know
- Other

In the last 12 months, approximately how many times have you administered a flea treatment to your cat?

- 1
- 2
- 3
- 4
- 5
- 6
- 7
- 8
- 9
- 10
- 11
- 12
- Don't know

Where do you usually purchase flea products for your cat?

- From a veterinary practice

- From a petshop
- From a supermarket
- Online
- Cat show event or similar
- Other type of shop - pharmacy/agricultural/equestrian/other
- None of the above
- Don't know

If you use a spot-on flea product on your cat, do you stroke your cat in the 24 hours after administering flea treatment? (Skip if not applicable)

- Yes
- No
- Don't know

In the last 12 months, have you seen or suspected any side effects to flea treatments in your cat?

- Yes
- No
- Maybe
- Cats (general)

In the last 12 months, have you suspected that your cat has fleas?

- Yes
- No
- Don't know

In the last 12 months, have you seen fleas on your cat or in your household?

- Yes
- No
- Don't know

Monthly occurrence of fleas (cats)

Approximately what month/s of the year did you see fleas on your cat or in your household?

- ☐ January
- ☐ February
- ☐ March
- ☐ April
- ☐ May
- ☐ June
- ☐ July
- ☐ August
- ☐ September
- ☐ October
- ☐ November
- ☐ December
- ☐ Don't know

Ticks

In the last 12 months, have you seen ticks on your cat?

- ○ Yes
- ○ No
- ○ Don't know

Monthly occurrence of ticks (cats)

Approximately what month/s of the year did you see ticks on your cat?

- ○ January
- ○ February
- ○ March
- ○ April
- ○ May
- ○ June
- ○ July
- ○ August
- ○ September

- October
- November
- December
- Don't know

Cats (general) part 2

In the last 12 months, approximately how many times have you treated your cat for worms?

- None
- 1
- 2
- 3
- 4
- 5
- 6
- 7
- 8
- 9
- 10
- 11
- 12
- Don't know

Where does your cat sleep? Select all that apply

- In their own bed
- On the furniture
- On a family member's bed
- On the floor
- Outside building/structure
- Other/ don't know

Where is your cat's most regular sleeping place? Select one

- In their own bed

- On the furniture
- On a family member's bed
- On the floor
- Outside structure/ building
- Other / don't know

Approximately how frequently do you wash your cat's bedding?

- Once a week or more
- Every 2 weeks
- Every month
- Every 3 months
- Every 6 months
- Once a year
- Never
- Don't know

Approximately how often does your cat sit on your lap?

- Every day
- Every 2 days
- Every week
- Every month
- Every 6 months
- Once a year
- Less than once a year
- Never
- Don't know

Has your cat gone outdoors in the last 12 months?

- Yes
- No
- Don't know

On a scale of 0-10, with 0 being 'never' and 10 being 'always', how often does your cat use a litter tray? 0

- 1
- 2
- 3
- 4
- 5
- 6
- 7
- 8
- 9
- 10
- Don't know

If your cat uses a litter tray, how do you dispose of the waste litter? Select all that apply. (Skip if not applicable)

- Throw in the bin
- Flush down the toilet
- Other/don't know

Does your cat hunt?

- Yes
- No
- Don't know

General flea questions

Do you have a regular subscription for flea/tick treatment?

- Yes
- No
- Don't know

In the last 12 months, have you sprayed your house for fleas?

- Yes
- No

- Don't know

Is your pet registered with a veterinarian?

- Yes
- No
- Don't know

Veterinary advice

What advice has been given by your veterinarian regarding flea/tick treatment?

- Regular preventative flea/tick treatment throughout the year
- Regular preventative flea/tick treatment in warmer months only
- Treat only if fleas/ticks are seen
- Treat only if fleas/ticks are seen, your pet has a history of being prone to flea/tick infestations, or suffers from skin allergies
- Other advice given
- No advice given
- Don't know

Do you follow your veterinarian's advice regarding frequency of flea/tick treatments? (Skip if not applicable)

- Yes
- No

Owner information

What is your age category?

- 18-24
- 25-34
- 35-44
- 45-54
- 55-65
- Over 65

What is your gender?

- ○ Female
- ○ Male
- ○ Prefer not to say
- ○ Other:

In which region of the UK do you live?

- ○ East
- ○ East Midlands
- ○ London
- ○ North East
- ○ North West
- ○ Northern Ireland
- ○ Scotland
- ○ South East
- ○ South West
- ○ Wales
- ○ West Midlands
- ○ Yorkshire And The Humber

Please enter the first half of your postcode. This will help to shed light on geographic patterns of flea product use.

Which of these best describes your location?

- ○ Urban
- ○ Suburban
- ○ Rural
- ○ Equally urban/suburban and rural

I am a:

- ○ Veterinarian
- ○ Veterinary Nurse

- Veterinary SQP (Suitably qualified Person)
- None of the above

For how long have you owned dogs and/or cats?

- Less than one year
- 1 - 2 years
- 2 - 5 years
- 5 - 10 years
- Over 10 years

On a scale of 0-10, with 0 being "not at all concerned" and 10 being "extremely concerned", how concerned are you about environmental issues? 0

- 1
- 2
- 3
- 4
- 5
- 6
- 7
- 8
- 9
- 10
- Don't know

On a scale of 0-10, with 0 being "not at all concerned" and 10 being "extremely concerned", how concerned are you about the potential environmental impact of pet flea products? 0

- 1
- 2
- 3
- 4
- 5
- 6
- 7

- 8
- 9
- 10
- Don't know

Where did you find out about this survey?

Powered by Qualtrics
